# Racial Differences in Glucose Handling and Function With Obesity Reduction: Preliminary Findings

**DOI:** 10.1093/geroni/igaa057.471

**Published:** 2020-12-16

**Authors:** Michael Borack, Marshall Miller, Jamie Rincker, Shelley McDonald, Kathyrn Starr, Connie Bales

**Affiliations:** 1 Duke University, Durham, North Carolina, United States; 2 Duke University, Efland, North Carolina, United States; 3 Duke University School of Medicine, Durham, North Carolina, United States

## Abstract

Blacks have higher rates of obesity and are twice as likely to develop diabetes as non-Hispanic whites. Obesity reduction can improve metabolic health, but physical function and glucose handling may be threatened by concomitant loss of muscle mass. These preliminary findings from a 4-mo. randomized controlled trial assess the racial differences in glucose handling and physical function in obese, older adults with prediabetes (Fasting Plasma Glucose (FPG) ≥95<126 mg/dL or HbA1c 5.7-6.4%) following obesity reduction. At 4 mo. endpoint, participants (n = 31; age = 68.1±5.4 years, BMI =36.0±4.7 kg/m2) had reduced (p<0.05) body weight in both Blacks (5.1%) and Whites (4.1%); HbA1c levels were also reduced (Blacks = -0.3 ±0.3; Whites = -0.1±0.3) with no difference by race. However, FPG was reduced for Blacks compared to Whites (-7.9±9.5 vs. -2.8±6.2 mg/dL; p<0.05). Short Physical Performance Battery (SPPB) score was lower for Blacks than Whites at both baseline (9.8±1.5 vs 10.9±1.2; p<0.05) and 4 mo. (10.17±1.4 vs 11.21±1.3; p<0.05), respectively. A trend towards improvement (p=0.08) in meters walked in 6 minutes was present in both Blacks (13.3±60.8) and Whites (20.0±36.3) with no between-group difference. Interestingly, at baseline, 41% of participants said they modified their behaviors due to a fear of falling despite having a mean SPPB score of 10.3±1.5. Following the intervention, fear of falling was reduced, with 35% of the participants reporting this behavior. Our findings illustrate that modest weight loss improves glucose handling, physical function and perceived fall risk for both Black and White older adults with prediabetes.

